# Identification of the Bok Interactome Using Proximity Labeling

**DOI:** 10.3389/fcell.2021.689951

**Published:** 2021-05-31

**Authors:** Laura M. Szczesniak, Caden G. Bonzerato, Richard J. H. Wojcikiewicz

**Affiliations:** Department of Pharmacology, SUNY Upstate Medical University, Syracuse, NY, United States

**Keywords:** Bcl-2 related ovarian killer, B-cell lymphoma 2 (Bcl-2) family, proximity labeling, myeloid-cell leukemia 1, apoptosis

## Abstract

The function of the Bcl-2 family member Bok is currently enigmatic, with various disparate roles reported, including mediation of apoptosis, regulation of mitochondrial morphology, binding to inositol 1,4,5-trisphosphate receptors, and regulation of uridine metabolism. To better define the roles of Bok, we examined its interactome using TurboID-mediated proximity labeling in HeLa cells, in which Bok knock-out leads to mitochondrial fragmentation and Bok overexpression leads to apoptosis. Labeling with TurboID-Bok revealed that Bok was proximal to a wide array of proteins, particularly those involved in mitochondrial fission (e.g., Drp1), endoplasmic reticulum-plasma membrane junctions (e.g., Stim1), and surprisingly among the Bcl-2 family members, just Mcl-1. Comparison with TurboID-Mcl-1 and TurboID-Bak revealed that the three Bcl-2 family member interactomes were largely independent, but with some overlap that likely identifies key interactors. Interestingly, when overexpressed, Mcl-1 and Bok interact physically and functionally, in a manner that depends upon the transmembrane domain of Bok. Overall, this work shows that the Bok interactome is different from those of Mcl-1 and Bak, identifies novel proximities and potential interaction points for Bcl-2 family members, and suggests that Bok may regulate mitochondrial fission via Mcl-1 and Drp1.

## Introduction

The Bcl-2 family mediates the intrinsic apoptosis pathway through the coordinated actions of pro- and anti-apoptotic proteins (Kale et al., 2018). The pro-apoptotic proteins include Bax and Bak, which mediate the release of cytochrome c from mitochondria via mitochondrial outer membrane permeabilization (MOMP), an effect opposed by the anti-apoptotic proteins Bcl-2, Mcl-1, and Bcl-x_L_. Pro-apoptotic sensitizer proteins, including Bad and Noxa, bind the anti-apoptotic proteins to prevent inhibition of apoptosis, while pro-apoptotic activator proteins, such as Bid and Bim, bind and activate Bax and Bak to facilitate MOMP. Many “non-apoptotic” roles for Bcl-2 family members have also been identified, including regulation of mitochondrial dynamics, Ca^2+^ homeostasis, and autophagy (Chong et al., 2020).

Bcl-2 related ovarian killer (Bok) was initially categorized as a pro-apoptotic Bcl-2 family member that can trigger MOMP (Hsu et al., 1997; Llambi et al., 2016), but many recent studies have also identified non-apoptotic functions (Ke et al., 2012; D’Orsi et al., 2016; Naim and Kaufmann, 2020; Shalaby et al., 2020). For instance, Bok “knock-out” (KO) from mouse embryonic fibroblasts (MEFs) causes mitochondrial fragmentation, which can be rescued by re-introduction of Bok (Schulman et al., 2019). This phenotype is intriguing, given that Bok is predominantly endoplasmic reticulum (ER)-localized (Echeverry et al., 2013) and constitutively bound to inositol 1,4,5-trisphosphate receptors (IP_3_Rs) (Schulman et al., 2013, 2016), which are tetrameric channels that release Ca^2+^ from ER stores. Bok has also been reported to protect IP_3_Rs from proteolysis (Schulman et al., 2013), mediate ER stress-induced apoptosis (Carpio et al., 2015), and positively regulate uridine metabolism (Srivastava et al., 2019).

Proximity-dependent biotin identification (BioID) was developed after the discovery that a point mutation, R118G, in the *Escherichia coli* biotin ligase protein BirA created a promiscuous ligase that could biotinylate proteins within an approximately 20 nm radius *in situ*, after the addition of exogenous biotin (Roux et al., 2012). Since the inception of BioID, multiple iterative modifications have been made to the original biotin ligase, the most recent being TurboID, which contains 16 mutations, permitting efficient biotin labeling *in situ* in as little as 15 min (Branon et al., 2018).

Here we show using TurboID that the Bok interactome is wide-ranging, but importantly, contains numerous ER and mitochondrial proteins, including mediators of mitochondrial fission, proteins involved in ER-plasma membrane (PM) contact, and Mcl-1. Further, we show that Bok and Mcl-1 interact physically and functionally and that the interactomes for Bok, Bak, and Mcl-1 are distinct, but overlap somewhat. These results shed light on the cellular roles of Bok and other Bcl-2 family members.

## Materials and Methods

### Materials

**FIGURE 1:**
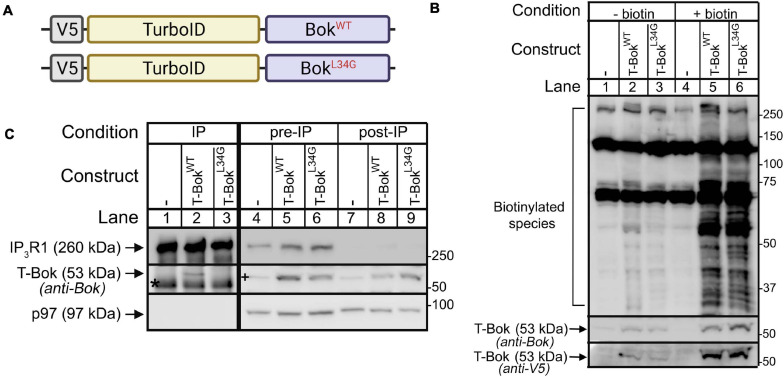
TurboID-Bok construct characterization. **(A)** V5-TurboID-Bok fusion constructs, T-Bok^WT^ and T-Bok^L34G^. **(B)** Immunoblot for biotin-labeled species (detected with streptavidin/anti-streptavidin) in lysates from Bok KO HeLa cells transfected as indicated to express T-Bok^WT^ or T-Bok^L34G^, without or with 2 h media supplementation with 50 μM biotin. Immunoreactivity of T-Bok constructs was assessed with either anti-Bok or anti-V5 (middle and lowest panels, respectively). **(C)** Anti-IP_3_R1/IP_3_R3 IP (lanes 1–3) and lysates (either pre- or post-IP; lanes 4–9) from Bok KO HeLa cells transfected as indicated, probed in immunoblots for the proteins indicated; p97 serves as a loading control. Co-migrating IgG heavy chain seen in the Bok probe of IPs is indicated by the asterisk. A 53kDa background band seen in the Bok probe of Bok KO cell lysates (lane 4) is indicated by the plus sign. Because Bok^L34G^ is relatively unstable (Schulman et al., 2016), to obtain equal expression, the amount of cDNA transfected for T-Bok^L34G^ was double that for T-Bok^WT^.

**FIGURE 2:**
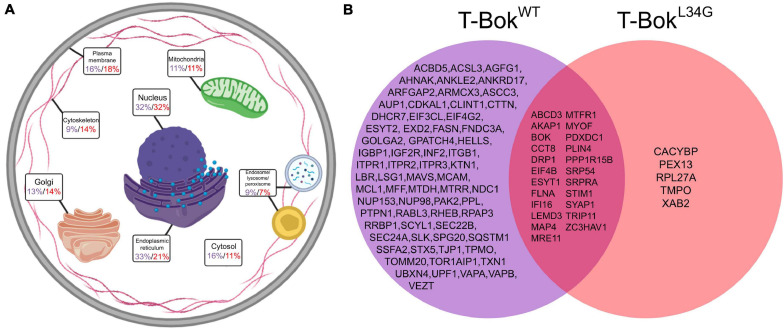
TurboID-Bok^WT^ and TurboID-Bok^L34G^ interactomes. **(A)** Localization of proteins identified by T-Bok^WT^ (purple) and T-Bok^L34G^ (red). Percentage values are the percent of proteins assigned to the specified subcellular compartment; as proteins were assigned to 1–3 compartments, percentages add to >100%. For further information, see Supplementary Tables 1, 2. **(B)** Comparison of proteins identified by T-Bok^WT^ and T-Bok^L34G^. The proteins shown were present in at least 6/7 and 4/5 independent experiments, respectively.

**FIGURE 3:**
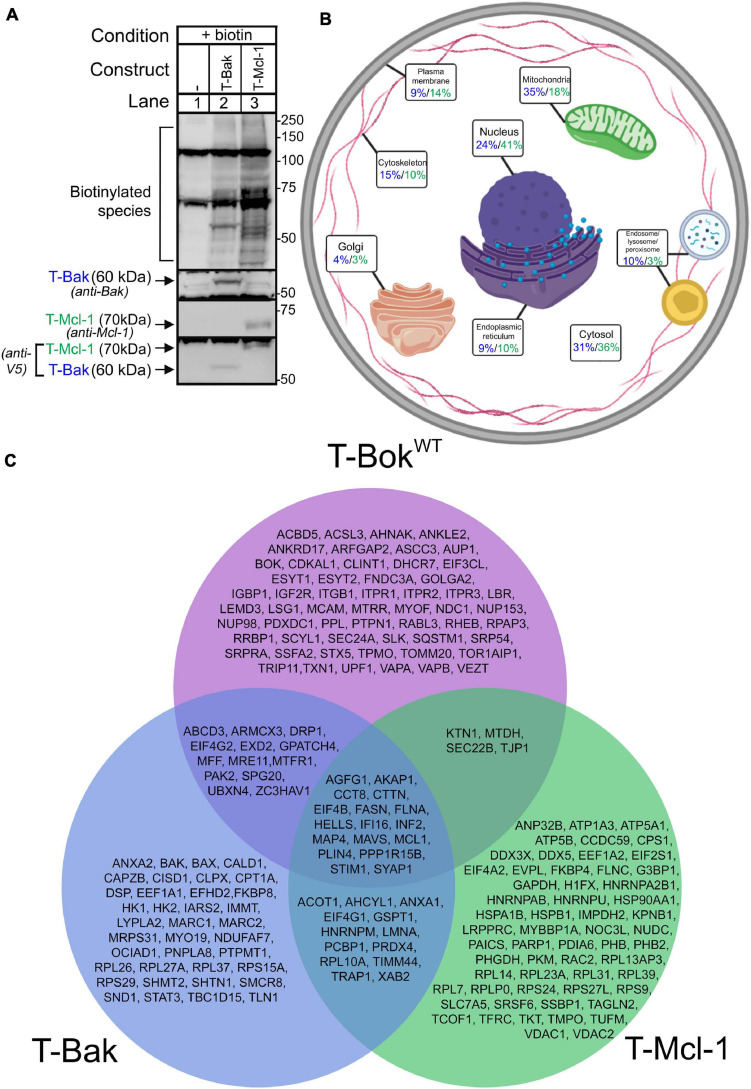
TurboID-Bak and TurboID-Mcl-1 interactomes. **(A)** Immunoblot for biotin-labeled species (detected with streptavidin/anti-streptavidin) in lysates from Bok KO HeLa cells transfected as indicated to express T-Bak or T-Mcl-1, without or with 2 h media supplementation with 50 μM biotin. Immunoreactivity of TurboID constructs was assessed with either anti-Bak, anti-Mcl-1, or anti-V5 (2nd-4th panels, respectively). **(B)** Localization of proteins identified by T-Bak (blue) and T-Mcl-1 (green). Percentage values are the percent of proteins assigned to the specified subcellular compartment; as proteins were assigned to 1–3 compartments, percentages add to >100%. For further information, see Supplementary Tables 3, 4. **(C)** Comparison of proteins identified by T-Bok^WT^, T-Bak, and T-Mcl-1. The proteins shown were present in at least 6/7, 2/3, and 2/3 independent experiments, respectively.

**FIGURE 4:**
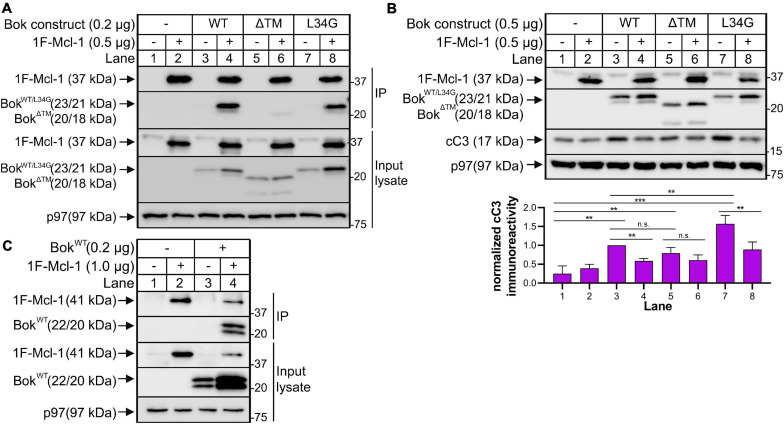
Overexpressed Mcl-1 and Bok interact with consequences on apoptotic signaling. **(A)** Bok KO HeLa cells were transfected to express 1F-mouse Mcl-1 and mouse Bok^WT^ and mutants as indicated for ∼18 h, and cell lysates and anti-Flag IPs were probed as indicated; p97 serves as a loading control. **(B)** Cleaved caspase-3 (cC3) immunoreactivity, visualized as an ∼17kDa band, was measured and quantified in Bok KO HeLa cells expressing 1F-mouse Mcl-1 and mouse Bok^WT^ and mutants as indicated for ∼18 h; a representative immunoblot is shown together with quantification of cC3 immunoreactivity using Image Lab software (mean ± SEM, *n* = 4). An unpaired *t*-test with Welch’s correction was used to determine significance; *p* < 0.005 is denoted by **, *p* < 0.0005 is denoted by ***, n.s. = not statistically significant. Data were graphed and analyzed using GraphPad Prism software. **(C)** Bok KO HeLa cells were transfected to express 1F-human Mcl-1 and human Bok^WT^ as indicated for ∼18 h, and cell lysates and anti-Flag IPs were probed as indicated; p97 serves as a loading control.

HeLa cells were maintained as described (Pearce et al., 2007). Antibodies raised in rabbits were: anti-Mcl-1 #D35A5 (for immunoblot), anti-Bcl-x_L_ #54H6, anti-Bax #2772, anti-Bcl-2 #50E3, anti-caspase-3 #9662, anti-pDrp1-616 #D9A1 and anti-pDrp1-637 #4867S (Cell Signaling Technology), anti-Bak #06-536 (Millipore), anti-IP_3_R1, anti-IP_3_R2 and anti-IP_3_R3 (for immunoprecipitation; IP) (Wojcikiewicz, 1995), anti-erlin2 (Pearce et al., 2007), and anti-Bok (Ke et al., 2012). Mouse monoclonal antibodies were: anti-Flag epitope (M2, Sigma), anti-IP_3_R3 #610313 (for immunoblot) and anti-Drp1 #611112 (BD Biosciences), anti-V5 epitope tag (GenScript), anti-Mcl-1 #RC13 (for IP) and anti-streptavidin #S10D4 (ThermoFisher), and anti-p97 (Research Diagnostics Inc.). Purified streptavidin was from BioLegend. PCR and Gibson reagents were from New England BioLabs. SDS-PAGE reagents were from Bio-Rad. Lipofectamine 2000 was from ThermoFisher. Cell culture dishes were from Corning. HRP-conjugated secondary antibodies and all other reagents not listed were from Sigma. Vectors encoding mouse and human Mcl-1 and Bok, and human Bak were kind gifts from Dr. T. Kaufmann (Echeverry et al., 2013). pCag-mouse Bok^WT^ (Schulman et al., 2016) and associated mutants used in Figure 4 (Bok^L34G^ and Bok^ΔTM^, which lacks amino acids 188-213) were created by PCR using existing primers (Schulman et al., 2013). BioRender was used to generate Figures 1A, 2A, 3B and Supplementary Figures 2, 3.

### Generation of Bok KO HeLa Cell Lines

The CRISPR-Cas9 system using the pCas-Guide-EF1a-GFP vector (#GE100018, OriGene) was used to generate Bok KO HeLa cells by targeting exon 2 (GTCTGTGGGCGAGCGGTCAA) or exon 4 (GCCCCGCGGCCACCGCATAC). Cells were transfected using Lipofectamine 2000, medium was changed after 24 h, and 48 h post-transfection, EGFP-expressing cells were selected by fluorescence-activated cell sorting and were seeded at one cell/well in a 96-well plate. Colonies were expanded and assessed for Bok immunoreactivity as described (Schulman et al., 2016). Multiple independent Bok KO cell lines for each exon target were used for all experiments.

### Generation of TurboID Fusion Proteins and Proximity Labeling

cDNAs were subcloned from vectors containing mouse Bok^WT^, mouse Bok^L34G^, mouse Mcl-1^WT^, and human Bak^WT^ (Echeverry et al., 2013; Schulman et al., 2016), and were ligated to the 3’ end (C terminus) of V5-TurboID in place of the stop codon (Addgene #107169) (Branon et al., 2018) using Gibson assembly, creating TurboID-Bok^WT^, TurboID-Bok^L34G^, TurboID-Mcl-1, and TurboID-Bak, respectively; the authenticity of all constructs was confirmed by DNA sequencing (Genewiz).

Bok KO HeLa cells in 10 cm dishes were transfected (0.325–1.25 μg cDNA/22.5 μL Lipofectamine), and ∼16 h later, medium was changed, cells were incubated with 50 μM biotin for 2 h, washed thrice with ice-cold PBS, and harvested with ∼500 μL ice-cold lysis buffer for IP as described (Schulman et al., 2016) or for streptavidin pull-down. To purify biotinylated proteins, lysates were incubated with 300μg/mL streptavidin C1 magnetic beads (ThermoFisher) for ∼16 h at 4°C, and beads were washed stringently with buffers containing SDS and detergents as described (Firat-Karalar and Stearns, 2015). All samples were re-suspended in gel loading buffer, boiled for 5 min, and subjected to SDS-PAGE. For pilot experiments (Figures 1B, 3A), lysates were transferred to nitrocellulose, and biotinylated species were detected by incubation with 100 ng/mL purified streptavidin (which binds to biotin with high affinity) for 1 h, anti-streptavidin for ∼16 h, and then developed similarly to other immunoblots. Once biotinylation was confirmed, purified biotinylated proteins were again subjected to SDS-PAGE until the dye front ran ∼2 cm, and lanes were excised for mass spectrometry (MS) analysis (described in Supplementary Methods).

### Analysis of MS Results

The MS data for each TurboID fusion protein underwent two stages of refinement (depicted in Supplementary Figure 3). In the first stage, for each experiment (i) proteins were excluded from further analysis if the *q* value > 0.01, (ii) keratin proteins were excluded, and (iii) proteins were included only if they were unique or if abundance was 5× increased in TurboID samples versus control samples (abundance = peptide spectrum match number divided by the total number of amino acids in the parent protein). In the second stage, lists of included proteins from a number (n) of independent experiments were compared, and proteins were considered to be “strongly labeled” if they were present in multiple (e.g., at least 6/7) lists. For each TurboID construct, lists of proteins after the first stage of refinement, plus the strongly labeled proteins, are shown in Supplementary Tables 1–4. Protein localization is described in the Supplementary Methods.

### Cell Lysis, IP and Immunoblotting

Lysates were prepared with ice-cold lysis buffer containing 1% Triton X-100, IPs were prepared with Protein A-Sepharose CL-4B beads (GE Healthcare), and lysates and washed IPs were subjected to SDS-PAGE and immunoblotting as described (Schulman et al., 2013, 2016).

## Results

### Bok Deletion in HeLa Cells Causes Mitochondrial Fragmentation

We selected HeLa cells for TurboID as they are relatively easy to transfect and have been used previously in proximity labeling studies (Roux et al., 2012). To facilitate analysis of the Bok interactome, we deleted endogenous Bok by CRISPR-Cas9 targeting of Bok exons 2 and 4, with no off-target effects on expression of other Bcl-2 family members or IP_3_Rs (Supplementary Figure 1A). Interestingly, imaging of these Bok KO cells indicated that Bok deletion causes mitochondrial fragmentation (Supplementary Figure 1B), with image quantification revealing significantly reduced mitochondrial particle length, area, and aspect ratio, while mitochondrial width was unchanged (Supplementary Figures 1C–F). Similar effects of Bok KO have been observed in MEFs (Schulman et al., 2019).

### TurboID-Bok as a Method to Identify the Bok Interactome

We performed TurboID experiments using wild-type Bok (Bok^WT^) and an L34G Bok mutant that cannot bind IP_3_Rs (Bok^L34G^) (Schulman et al., 2013) to determine if the proteins proximal to Bok are dependent on the interaction of Bok and IP_3_Rs. Both Bok^WT^ and Bok^L34G^ were fused to V5-tagged TurboID, creating TurboID-Bok^WT^ and TurboID-Bok^L34G^ (T-Bok^WT^ and T-Bok^L34G^, respectively, Figure 1A). TurboID was fused to the N- termini to minimize the possibility of mislocalization, since Bok is localized to the ER by its C-terminal transmembrane (TM) domain (Echeverry et al., 2013).

Expression of T-Bok^WT^ and T-Bok^L34G^ in Bok KO HeLa cells resulted in an exogenous biotin-dependent smear of biotinylated species (Figure 1B, lanes 5, 6). The two prominent bands at ∼130 and 70 kDa, seen in all lanes, were identified by MS analysis as the endogenously biotinylated proteins pyruvate carboxylase and propionyl-CoA carboxylase (Tong, 2013), respectively. It is also noteworthy that the immunoreactivity of the T-Bok constructs increased after the addition of biotin (Figure 1B, lanes 2–3 versus 5–6), consistent with previous findings that TurboID constructs are stabilized by exogenous biotin (Branon et al., 2018).

To determine how well the T-Bok constructs interact with IP_3_Rs, we examined their ability to co-IP with endogenous IP_3_Rs (Figure 1C). This showed, as expected, that T-Bok^WT^, but not T-Bok^L34G^, binds IP_3_Rs (lane 2 versus 3) (Schulman et al., 2013). Importantly, the difference between pre-IP versus post-IP lysates suggest that most of T-Bok^WT^ is associated with IP_3_Rs (lane 5 versus 8).

From cells incubated with biotin as in Figure 1B (lanes 4–6), biotinylated proteins were purified using streptavidin-coated beads followed by SDS-PAGE, trypsin digestion, and MS analysis (Supplementary Figure 2). The initial list of proteins obtained for each T-Bok sample underwent two stages of data refinement to remove non-specifically interacting proteins (Supplementary Figure 3). The first stage excluded any proteins that were also found in control (non-transfected) samples analyzed on the same day, and the second stage included proteins present only in multiple independent experiments; for T-Bok^WT^ and T-Bok^L34G^, only proteins present in at least 6/7 and 4/5 experiments, respectively, were included. These “strongly labeled” proteins were categorized for subcellular localization (Supplementary Tables 1, 2 and Figure 2A) and were compared to determine similarities and differences (Figure 2B). Interestingly, fewer proteins were identified by T-Bok^L34G^ than T-Bok^WT^ (28 versus 90 proteins, respectively), Bok was present on both lists due to self-biotinylation, and only T-Bok^WT^ labeled IP_3_Rs (Itpr1-3), indicating that the approach is valid.

T-Bok^WT^ was proximal to a broad array of proteins at different sites (Figure 2A), although ER and nuclear localizations were predominant (33 and 32% of labeled proteins, respectively). While the identification of ER proteins is to be expected due to Bok’s constitutive ER localization with IP_3_Rs (Echeverry et al., 2013; Schulman et al., 2016), the high number of nuclear proteins is surprising. However, proteins were assigned up to 3 locations, and many of the multi-located proteins included a nuclear assignment. Additionally, several of the nucleus-assigned proteins were nuclear membrane proteins, which is understandable given the contiguous nature of the ER and nuclear membrane (English and Voeltz, 2013). Also in the T-Bok^WT^ protein list were several PM (16%), cytosolic (16%), Golgi (13%), and mitochondrial proteins (11%).

More detailed consideration of proteins identified for T-Bok^WT^ (Figure 2B) revealed clusters of proteins known to regulate mitochondrial fission (Drp1, Mff, Inf2, Akap1, etc.) (Czachor et al., 2016; Kraus et al., 2021) and ER-PM contact sites (Itpr1-3, Stim1, Vapa, Vapb, etc.) (Murphy and Levine, 2016; Prole and Taylor, 2019). Drp1 is a GTPase well-known for being the main effector protein for mitochondrial fission (Kraus et al., 2021), Stim1 is an ER protein involved in store-operated Ca^2+^ entry (SOCE) and ER-PM junctions (Prole and Taylor, 2019), and Akap1 is a mitochondrial membrane scaffolding protein that regulates a variety of mitochondrial functions (Czachor et al., 2016). Notably, no Bcl-2 family members were labeled by T-Bok^WT^ aside from Mcl-1. Surprisingly, we did not detect uridine monophosphate synthetase, despite recent reports that Bok regulates uridine metabolism by enhancing its activity (Srivastava et al., 2019), or key proteins involved in mitochondrial fusion (e.g., Mfn1/2), despite evidence that Bok can regulate fusion rate (Schulman et al., 2019).

The result that T-Bok^WT^ strongly labeled three times as many proteins as T-Bok^L34G^ was initially perplexing. However, it is likely that T-Bok^L34G^ is rapidly turned over because it cannot interact with IP_3_Rs (Schulman et al., 2016), and this may impair biotin ligase activity. Indeed, to achieve comparable expression and biotinylation required using twice as much T-Bok^L34G^ cDNA than T-Bok^WT^ cDNA (Figures 1B,C). Nevertheless, the protein list for T-Bok^L34G^ overlapped significantly with that of T-Bok^WT^, suggesting that the mutant protein still localizes to the ER despite not binding to IP_3_Rs. A comparison of the two T-Bok constructs with a lower threshold for T-Bok^L34G^ (i.e., addition of proteins in 3/5 experiments, Supplementary Figure 4) identified more proteins that were also present in the T-Bok^WT^ list, including more of the proteins involved in mitochondrial fission (Inf2 and Mff). Overall, the interactome of T-Bok^L34G^ has some similarities to that of T-Bok^WT^, but also major differences. Some of these differences may result from localization of T-Bok^WT^ to IP_3_Rs, but likely also reflect the marked differences in T-Bok^WT^ and T-Bok^L34G^ stability.

### TurboID-Bak and TurboID-Mcl-1 Interactomes

To assess T-Bok^WT^ proximity labeling specificity and better understand other Bcl-2 family members, we generated TurboID constructs for Bak, which localizes predominantly to mitochondria, and Mcl-1, which also localizes to mitochondria, but is also found at the ER and in the cytosol (Kale et al., 2018). Both TurboID-Bak (T-Bak) and TurboID-Mcl-1 (T-Mcl-1) expressed and induced biotinylation similarly to T-Bok^WT^ (Figure 3A).

TurboID was performed in Bok KO HeLa cells to allow for direct comparison with T-Bok^WT^ results. The possible impact of the presence of endogenous Mcl-1 and Bak in these cells (Supplementary Figure 1A) on labeling was not examined in this study. Localization of strongly labeled proteins (Figure 3B and Supplementary Tables 3, 4) demonstrates that both T-Bak and T-Mcl-1 did not identify as many ER proteins as T-Bok^WT^ (9, 10, and 33%, respectively), as expected. In order of abundance, T-Bak identified mitochondrial (35%), cytosolic (31%), and nuclear proteins (24%), whereas T-Mcl-1 identified nuclear (41%), cytosolic (36%), and mitochondrial proteins (18%). Again, as expected, both T-Bak and T-Mcl-1 labeled more mitochondrial proteins than T-Bok^WT^.

Comparison of the protein lists for the three Bcl-2 family proteins (Figure 3C) demonstrated that each interactome is quite distinct, although there was significant overlap. In particular, Inf2 and Mavs, which are involved in mitochondrial fission (Kraus et al., 2021) and fusion (Koshiba et al., 2011), respectively, were present in all lists, as were Akap1 and Stim1. Also, both T-Bok^WT^ and T-Bak labeled Mcl-1, which is consistent with studies indicating that Bok (Hsu et al., 1997) and Bak (Cuconati et al., 2003) can physically interact with Mcl-1.

The protein lists for T-Bak and T-Bok^WT^ showed some overlap, and notably, the mitochondrial fission proteins Mff and Mtfr1 were common to both. Many of the proteins unique to the T-Bak list were mitochondrial proteins, and not surprisingly, Bax was among them (Kale et al., 2018). T-Mcl-1 showed only minor overlap with T-Bok^WT^, and uniquely labeled Vdac1/2, consistent with findings that Mcl-1 physically interacts with Vdac and can facilitate Vdac-dependent mitochondrial Ca^2+^ uptake (Huang et al., 2014). Overall, the discrete proximity labeling patterns for T-Bok^WT^, T-Bak, and T-Mcl-1 validate the TurboID approach, and comparison of the three protein lists reflects the complexity of the Bcl-2 family network and identifies potential novel interactions.

### Analysis of the Bok-Mcl-1 Interaction

Since T-Bok^WT^ labeled Mcl-1 but no other Bcl-2 family member (Figure 2), we wondered whether this signified mere proximity between Bok and Mcl-1, or whether they interact directly. Interestingly, when co-expressed, Bok^WT^ did co-IP with 1F-Mcl-1 (Mcl-1 with an N-terminal Flag tag), and 1F-Mcl-1 increased Bok immunoreactivity (Figure 4A, lane 4), suggesting that the interaction stabilizes Bok. This was still observed with Bok^L34G^ (lane 8), indicating that it was not mediated by the BH4 domain of Bok that is critical for the interaction with IP_3_Rs (Schulman et al., 2013), but was markedly reduced for Bok^ΔTM^ (lane 6), indicating that Bok localization to membranes is important for the interaction, or that it is directly mediated by the Bok TM domain itself (Lucendo et al., 2020). Under the same conditions, endogenous Bok did not co-IP with endogenous Mcl-1 (Supplementary Figure 5), as noted in some other studies (Echeverry et al., 2013; Schulman et al., 2016), indicating that the Bok-Mcl-1 interaction is relatively weak and is only detectable upon protein overexpression. Nevertheless, the interaction between exogenous Bok^WT^ and 1F-Mcl-1 has functional significance, as Bok^WT^-mediated increases in cleaved caspase-3 (cC3), were suppressed significantly by 1F-Mcl-1 (Figure 4B, lanes 3 versus 4). Interestingly, Bok^ΔTM^ increased cC3 levels similarly to Bok^WT^, but the effect of Bok^ΔTM^ was not significantly suppressed by 1F-Mcl-1 (lanes 5 versus 6), consistent with their much weaker physical interaction (Figure 4A, lane 6). Further, Bok^L34G^ induced significantly more cC3 than Bok^WT^ (Figure 4B, lanes 3 versus 7), presumably because Bok^L34G^ is not sequestered by IP_3_Rs, although Mcl-1 suppressed the response (lanes 7 versus 8). Overall, these data indicate that the proximity labeling approach can identify fleeting protein-protein interactions of functional significance that might not be detectable by conventional (e.g., co-IP) analysis of endogenous proteins, and that Mcl-1 likely mediates or modulates the effects of Bok. It should be noted that in these studies, we utilized mouse Bok and Mcl-1 expressed in human HeLa cells. However, mouse and human Bok and Mcl-1 amino acid sequences are 95% and 76% identical, respectively, and human Bok and Mcl-1 co-IP like their mouse counterparts when over-expressed in Bok KO HeLa cells (Figure 4C), indicating that the mouse and human proteins behave identically in the HeLa cell context.

### Bok Deletion in HeLa Cells Does not Alter Drp1 Phosphorylation Status or Ca^2+^ Mobilization

Since Drp1 is a crucial mediator of mitochondrial fission (Kraus et al., 2021) and was strongly labeled by T-Bok^WT^ (Figure 2), we sought to determine if Bok regulates Drp1 activity, which can be measured through Drp1 phosphorylation (pDrp1). pDrp1^S616^ is associated with increased mitochondrial fission, whereas pDrp1^S637^ is associated with decreased mitochondrial fission (Kraus et al., 2021). Using validated antibodies, we examined pDrp1 levels among HeLa cell lines and found that pDrp1^S616^ and pDrp1^S637^ levels were not substantially changed by Bok deletion (Supplementary Figure 6). Likewise, given that several proteins related to intracellular Ca^2+^ signaling at the ER or at ER-PM contact sites were labeled by T-Bok^WT^, we sought to determine whether Bok KO altered Ca^2+^ mobilization. However, WT and Bok KO HeLa cells responded essentially identically to trypsin (Supplementary Figure 7), indicating that Bok KO does not affect Ca^2+^ signaling.

## Discussion

Proximity labeling was developed to identify the interactome for a given protein by labeling transient interactions and nearby proteins, providing an alternative to the traditional co-IP or co-purification approaches that reveal only the highest affinity protein-protein interactions (Roux et al., 2012). Here we show that TurboID can efficiently and specifically identify the interactomes for Bok, Mcl-1, and Bak. T-Bok-labeled proteins are predominantly found in the ER, consistent with Bok’s reported subcellular localization. In contrast, T-Bak and T-Mcl-1 mostly labeled mitochondrial and nuclear proteins, respectively, consistent with Bak’s known localization to the mitochondrial membrane, and a more mixed distribution for Mcl-1 (Kale et al., 2018).

While the proteins labeled by T-Bok^WT^ were predominantly ER-residents, protein groups in other locations were also identified, indicating a role for Bok at the interface of the ER and other organelles. As Bok deletion causes mitochondrial fragmentation, we particularly focused on mitochondrial proteins. Mitochondria-ER contacts (MERCs), sometimes referred to as mitochondrial-associated membranes (MAMs) when isolated in biophysical protocols, are transient microdomains where ER and mitochondria come within 10-80 nm of each other (Giacomello and Pellegrini, 2016). Several studies report that MERCs are essential for numerous signaling processes, including Ca^2+^ transfer, lipid trafficking/metabolism, and regulation of cell death or survival (Perrone et al., 2020), and interestingly, a recent study suggests that Bok is integral to the stability of MERCs/MAMs (Carpio et al., 2021). However, aside from IP_3_Rs, T-Bok did not label any of the proteins reported to be important in the coupling of the ER to mitochondria at MERCs/MAMs, including Vdac1, Grp75, or Mfn2 (Perrone et al., 2020). Rather, T-Bok identified several proteins important for mitochondrial fission, including Drp1, Mff, Akap1, and Inf2 (Czachor et al., 2016; Kraus et al., 2021). This suggests that the role of Bok at the interface of ER and mitochondria is not to maintain MERC/MAM structure or function, but rather to regulate mitochondrial fission. This notion is consistent with findings that the ER is highly involved in mitochondrial fission (Perrone et al., 2020), and that ER projections can wrap around mitochondria to mediate the division process (Friedman et al., 2011).

Could an inhibitory effect of Bok on key fission mediators, such as Drp1, explain the mitochondrial fragmentation seen in Bok KO cells? This is a distinct possibility, since although we were unable to see an effect of Bok KO on Drp1 levels or phosphorylation (a measure of Drp1 activity), Drp1 function is regulated by several post-translational modifications aside from phosphorylation (Chang and Blackstone, 2010), and other fission mediators (e.g., Inf2, Mff), could also be regulated by interfacing with Bok. Further, as indicated below, Mcl-1 could mediate effects of Bok on mitochondrial fission.

That the protein list for T-Bok^L34G^, which does not bind IP_3_Rs, was considerably shorter than that for T- Bok^WT^ and other TurboID-fusion proteins, is likely explained by the instability of T-Bok^L34G^ (Schulman et al., 2016); presumably, the rapid turnover of T- Bok^L34G^ impairs its ability to elicit significant biotinylation. Unfortunately, this unexpected finding made it impossible to accurately assess the effect of localization to IP_3_Rs on the T-Bok^WT^ interactome. Nevertheless, it is interesting that almost all of the proteins strongly labeled by T-Bok^L34G^ were also labeled by T-Bok^WT^, indicating that T-Bok^L34G^ is localized similarly to T-Bok^WT^. This is consistent with the ability of Bok^L34G^ to restore normal mitochondrial morphology when introduced into Bok KO cells (Schulman et al., 2019).

The overlap in proteins labeled by T-Bok, T-Bak, and T-Mcl-1 is intriguing and may open new research avenues. For example, the scaffolding protein Akap1, which modulates numerous signaling pathways at the mitochondrial surface (Czachor et al., 2016), was labeled by T-Bok, T-Bak, and T-Mcl-1, suggesting that it may be a general mediator of Bcl-2 family-related processes at the mitochondrial membrane. Likewise, all 3 proteins labeled Stim1, an ER membrane protein involved in ER-PM coupling for SOCE (Prole and Taylor, 2019). To the best of our knowledge, interactions between Stim1 and Bok, Bak, or Mcl-1 have not been previously reported, and although we did not detect an effect of Bok KO on Ca^2+^ signals that include SOCE, Bcl-2 does regulate SOCE (Vanden Abeele et al., 2002; Chiu et al., 2018), suggesting that many Bcl-2 family members may regulate this pathway. Lastly, both T-Bok and T-Bak identified Mcl-1, and Mcl-1 was the only Bcl-2 family member identified by T-Bok. This is consistent with the widespread distribution of Mcl-1 (Kale et al., 2018) and indicates that analysis of the Bok-Mcl-1 interaction may be a fruitful approach to solving the puzzle of how Bok acts within the cell.

Indeed, we were able to show that Bok and Mcl-1 interact physically, albeit only when overexpressed, and that this binding has functional consequences, since Mcl-1 inhibited Bok-mediated apoptotic signaling. These finding are broadly consistent with those of others (Hsu et al., 1997; Stehle et al., 2018; Lucendo et al., 2020), but with some significant differences. In particular, our findings that Bok^ΔTM^ mediates apoptotic signaling similarly to Bok^WT^ contradict a recent study (Stehle et al., 2018) indicating that the TM domain of Bok is required for apoptosis, although this could be accounted for by the different experimental systems used. The Bok-Mcl-1 interaction also provides a potential mechanism for Bok to regulate mitochondrial morphology, since Mcl-1 regulates mitochondrial fission, at least in part, by acting through Drp1 (Moyzis et al., 2020). As we find that T-Bok^WT^ strongly labels both Mcl-1 and Drp1, it is possible that Bok inhibits mitochondrial fission rate by modulating the action of Mcl-1. Thus, upon Bok KO, fission rate would be accelerated, explaining the mitochondrial fragmentation observed in Bok KO MEFs (Schulman et al., 2019) and Hela cells (Supplementary Figure 1). Interestingly, in our previous studies on the mechanism of Bok KO-induced mitochondrial fragmentation we could not measure mitochondrial fission rate directly, but found that Bok KO inhibits fusion rate. Since T-Bok identified mitochondrial fission mediators but not fusion mediators, we speculate that the effect of Bok KO on fusion rate may be an adaptation to a direct effect of Bok KO on fission mediators and fission rate.

It is important to note that the proteins identified by TurboID constructs reveal proximity, but not necessarily functional interactions. As a “shotgun” approach to the study of possible protein-protein interactions, deriving meaning from proximity labeling still requires functional studies, such as those we performed with Bok and Mcl-1. In that particular case, we were able to demonstrate functional consequences from the Bok-Mcl-1 interaction, but it is also inevitable that some, perhaps most, identified proteins will not interact physically, and other methods (e.g., CRISPR-Cas9-mediated protein KO) will be required to establish significance of proximity. Overall, we hope that this study serves to drive further research into Bok and Bcl-2 family interactions, with outcomes that will lead to a better understanding of Bok and cell physiology.

Going forward, it will be particularly interesting to determine if stably expressed Bcl-2 family proteins biotinylate more specifically than the transient expression method used in the present study, how the interactomes might change when apoptosis is triggered, how endogenous Mcl-1 and Bak impact the labeling seen with T-Mcl-1 and T-Bak, and how certain proteins strongly labeled by T-Bok might help explain the various putative roles of Bok. In particular, the interaction with anti-apoptotic Mcl-1 and thus the Bcl-2 family network may explain how manipulating Bok levels can have various effects on apoptotic signaling (Naim and Kaufmann, 2020; Shalaby et al., 2020), the identification of Drp1 and other fission mediators may explain how Bok can influence mitochondrial morphology (Schulman et al., 2019), and the identification of ER-PM and ER-Golgi junctional proteins (e.g., Stim1, Vapa and Vapb) suggest new possible roles for Bok in inter-organelle contact sites.

## Data Availability Statement

The authors acknowledge that the data presented in this study must be deposited and made publicly available in an acceptable repository, prior to publication. Frontiers cannot accept a manuscript that does not adhere to our open data policies.

## Author Contributions

LS performed, guided, and analyzed all the experiments shown and was the primary author of the manuscript. CB designed and implemented screening of HeLa Bok KO cell lines and assisted with image acquisition for Supplementary Figure 1B. RW conceived and coordinated the study with substantial editorial input into the manuscript. All authors reviewed the results and approved the final version of the manuscript.

## Conflict of Interest

The authors declare that the research was conducted in the absence of any commercial or financial relationships that could be construed as a potential conflict of interest.

## Abbreviations

BioID/TurboID: proximity-dependent biotin identification
Bok: Bcl-2-related ovarian killer
cC3: cleaved caspase-3
ER: endoplasmic reticulum
IP: immunoprecipitation
IP_3_R: inositol 1,4,5-trisphosphate receptor
KO: knock-out
MAM: mitochondrial-associated membrane
MEF: mouse embryonic fibroblast
MERC: mitochondria-ER contact site
MOMP: mitochondrial outer membrane permeabilization
MS: mass spectrometry
pDrp1: phosphorylated Drp1
PM: plasma membrane
SOCE: store-operated calcium entry
T-Bak: TurboID-Bak
T-Bok: TurboID-Bok
T-Mcl-1: TurboID-Mcl-1
TM: transmembrane.
