# Supplementary material for: Identification of the Bok Interactome Using Proximity Labeling
Source: Front Cell Dev Biol. 2021 May 31;9:689951. doi: 10.3389/fcell.2021.689951 (PMC8201613; doi:10.3389/fcell.2021.689951)
Supplement: Supplementary file 5 [file Data_Sheet_1.PDF]

Supplementary Figures

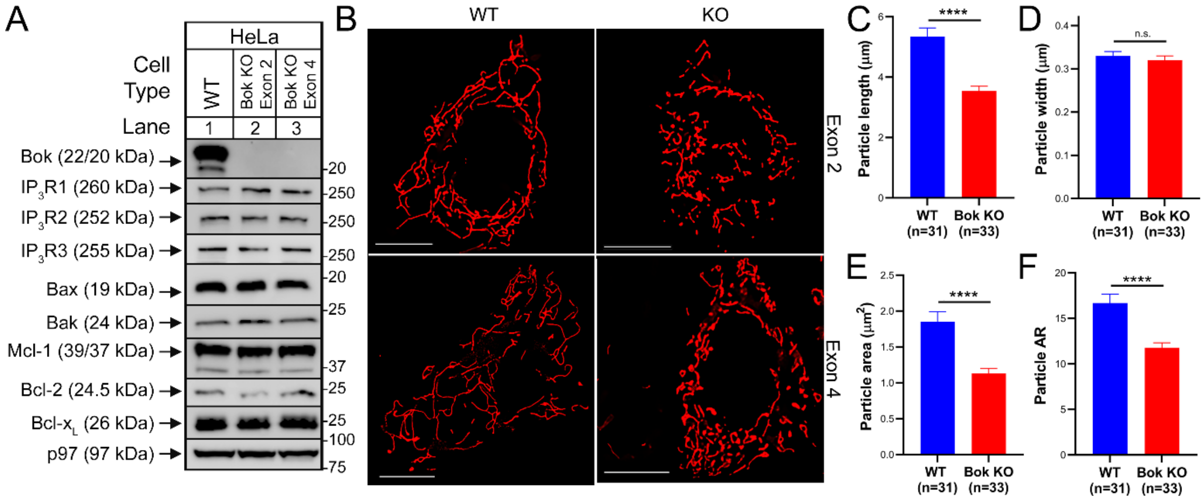

Supplementary Figure 1. Bok deletion causes mitochondrial fragmentation in HeLa cells. **(A),** Immunoblot demonstrating specific deletion of Bok in lysates from WT versus Bok KO HeLa cells, obtained by targeting exon 2 (lane 2) or exon 4 (lane 3), p97 serves as a loading control. **(B),** Representative confocal images of WT and Bok KO HeLa cells transiently expressing DsRed2-Mito (scale bar = 10µm). Cells were seeded onto 35 mm glass bottom dishes and the next day were transfected with polyethyleneimine as described[1] with a plasmid encoding DsRed2-Mito (Clontech #632421). ~24 h later, cells were imaged at 37°C/5% CO_2 with a Nikon C2 microscope equipped with a 60 × Plan Apo VC oil-immersion objective lens, using 543.5 nm excitation/585 nm emission. All images were subjected to 1 iteration of blind deconvolution using NIS-Elements software. **(C-F),** Quantified measurements of mitochondrial particle length, width, area and aspect ratio (AR = length divided by width) in WT and Bok KO HeLa cells obtained using NIS-Elements software. Quantitated data included WT and both exon 2 and exon 4-targeted cells and are expressed as mean ± SEM (n = the number of cells analyzed). An unpaired t-test was used to determine significance; p<0.0001 is denoted by ****, n.s. = not statistically significant. Data were graphed and analyzed using GraphPad Prism software.

References

[1] Schulman, J. J., Wright, F. A., Han, X., Zluhan, E. J., Szczesniak, L. M., and Wojcikiewicz, R. J. (2016) The Stability and Expression Level of Bok Are Governed by Binding to Inositol 1,4,5-Trisphosphate Receptors, *J Biol Chem 291*, 11820-11828.
