# Supplementary figures and images for: Identification of the Bok Interactome Using Proximity Labeling
Source: Front Cell Dev Biol. 2021 May 31;9:689951. doi: 10.3389/fcell.2021.689951 (PMC8201613; doi:10.3389/fcell.2021.689951)

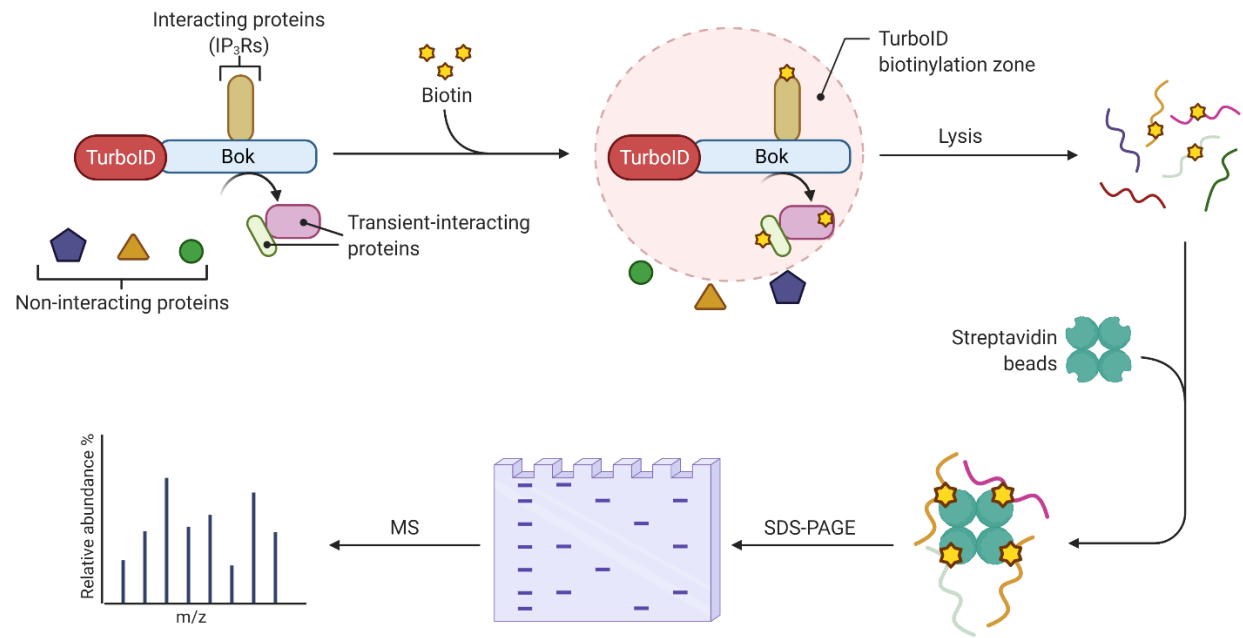

**Supplementary Figure 2.** Overview of TurboID experimental procedure.

Supplement: Supplementary file 6 [file Data_Sheet_2.PDF]
