# Supplementary material for: Identification of the Bok Interactome Using Proximity Labeling
Source: Front Cell Dev Biol. 2021 May 31;9:689951. doi: 10.3389/fcell.2021.689951 (PMC8201613; doi:10.3389/fcell.2021.689951)
Supplement: Supplementary file 7 [file Data_Sheet_3.PDF]

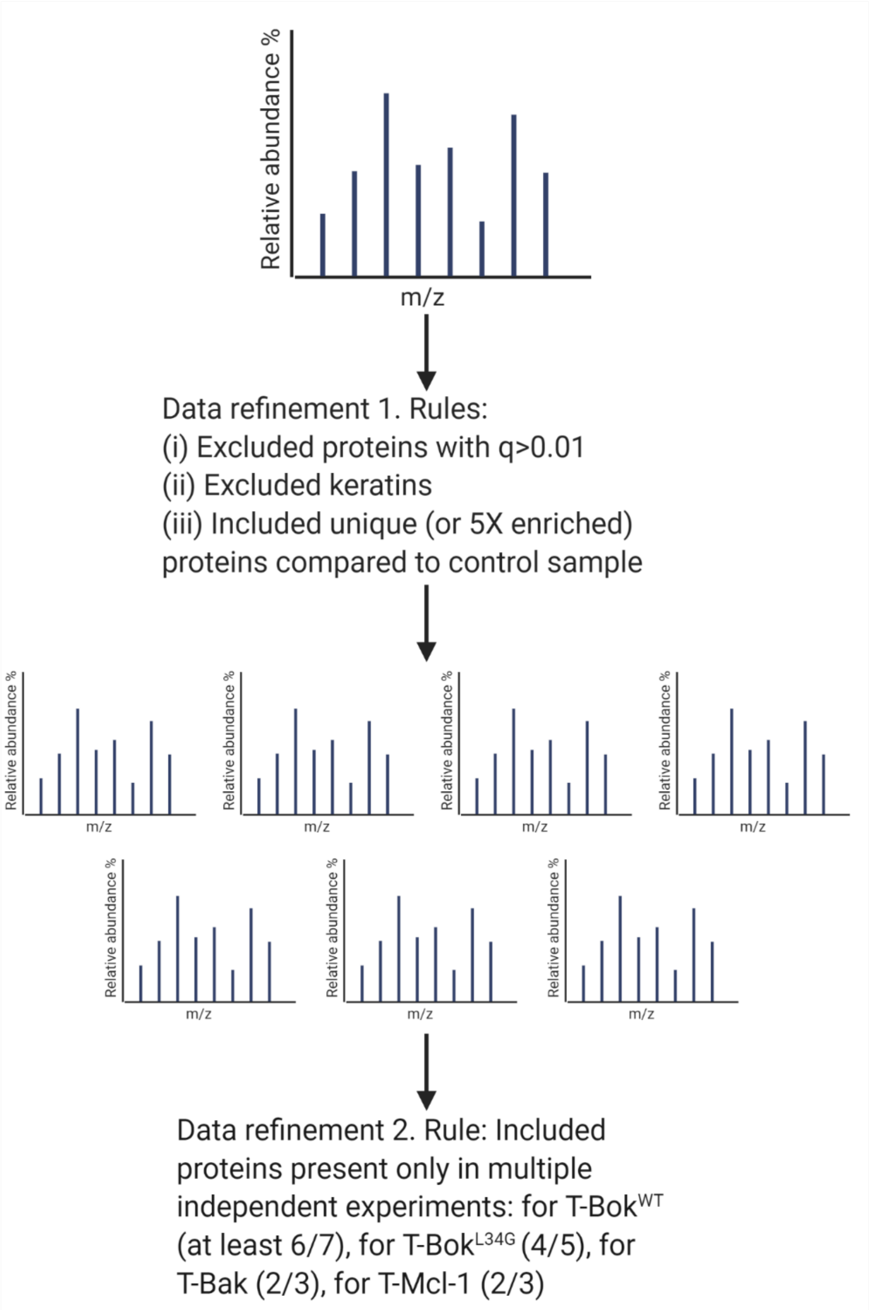

Supplementary Figure 3. Overview of data refinement of TurboID MS results. Data refinement 1 was implemented for each individual experiment with control and TurboID samples to exclude proteins binding to streptavidin-coated beads non-specifically: (i) proteins were excluded if q>0.01 (i.e. medium/low confidence identification), (ii) keratins were excluded, and (iii) proteins were included only when unique or if abundance was 5x increased in TurboID samples versus control samples. For data refinement 2, proteins present only in multiple data sets, or “strongly labeled” proteins, were included for further consideration. Due to the inherent variability in the TurboID procedure, proteins were considered as strongly labeled if present in *n-1* independent experiments.
