# Supplementary material for: Identification of the Bok Interactome Using Proximity Labeling
Source: Front Cell Dev Biol. 2021 May 31;9:689951. doi: 10.3389/fcell.2021.689951 (PMC8201613; doi:10.3389/fcell.2021.689951)
Supplement: Supplementary file 8 [file Data_Sheet_4.PDF]

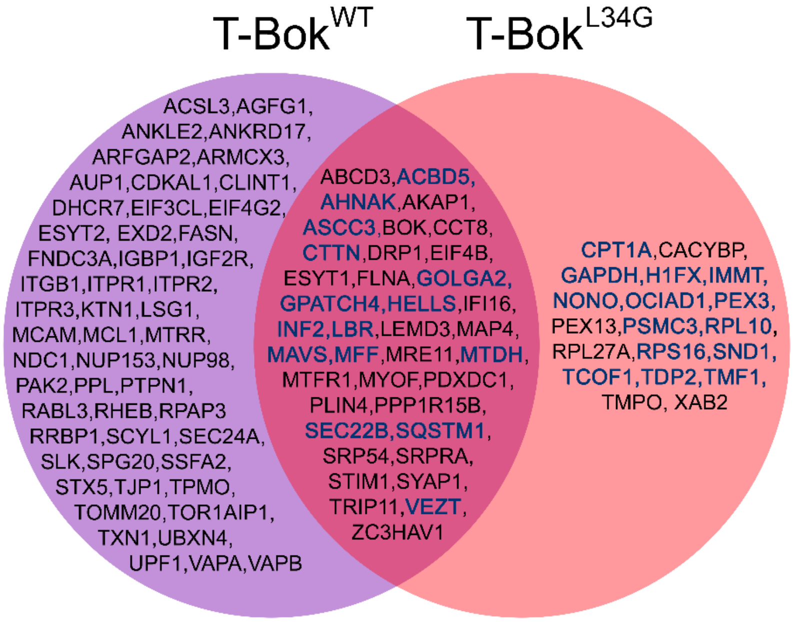

Supplementary Figure 4. Comparison of proteins identified by T-Bok^{WT} and T-Bok^{L34G} at lower stringency. Proteins labeled by T-Bok^{L34G} that are present in 3/5 independent experiments are blue and emboldened; all other proteins (black text) are the same as those shown in Fig. 2B.
