# Supplementary material for: Identification of the Bok Interactome Using Proximity Labeling
Source: Front Cell Dev Biol. 2021 May 31;9:689951. doi: 10.3389/fcell.2021.689951 (PMC8201613; doi:10.3389/fcell.2021.689951)
Supplement: Supplementary file 9 [file Data_Sheet_5.PDF]

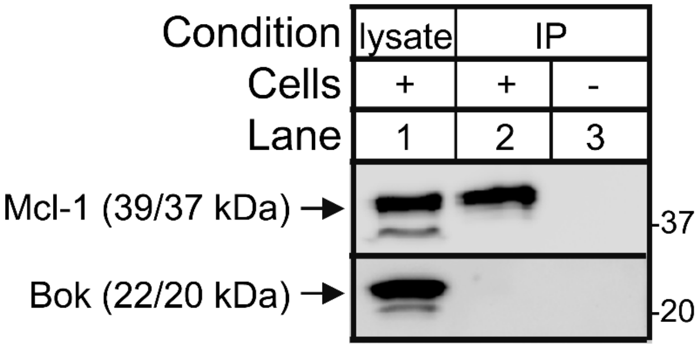

Supplementary Figure 5. HeLa cell lysate (lane 1) and anti-Mcl-1 IP (lane 2) and control IP (lane 3) were probed as indicated, showing that the Bok-Mcl-1 interaction is not readily detectable for endogenous proteins.
