# Supplementary material for: Identification of the Bok Interactome Using Proximity Labeling
Source: Front Cell Dev Biol. 2021 May 31;9:689951. doi: 10.3389/fcell.2021.689951 (PMC8201613; doi:10.3389/fcell.2021.689951)
Supplement: Supplementary file 10 [file Data_Sheet_6.PDF]

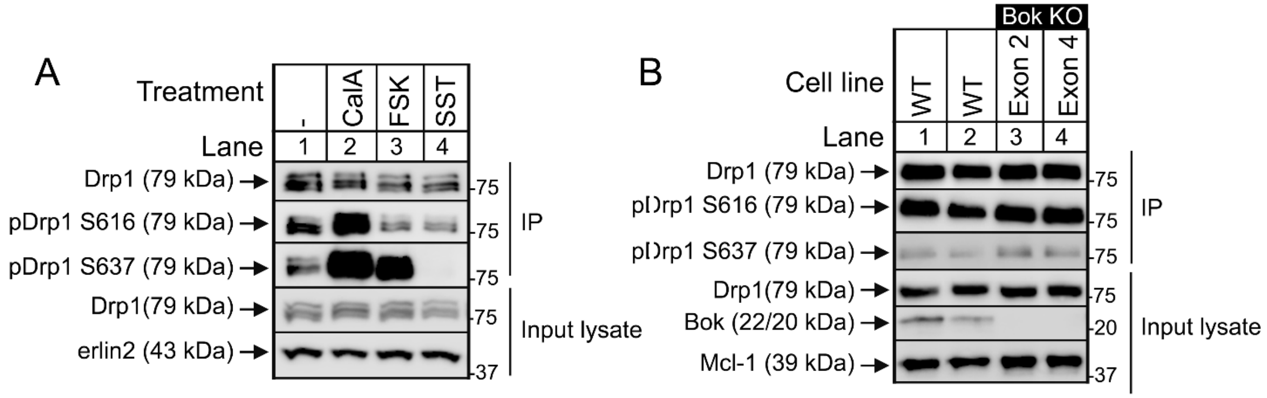

Supplementary Figure 6. Examination of pDrp1$^{S616/S637}$ levels in HeLa cells. **(A),** Validation of pDrp1-specific antibodies anti-pDrp1^{S616} and pDrp1^{S637}. HeLa cells were treated either with 50 nM calyculin A (CalA) for 30 min, 20 µM forskolin (FSK) for 30 min, or 1 µM staurosporine (SST) for 2 hours. Anti-Drp1 IPs and input lysates were probed as indicated; erlin2 serves as a loading control for the input lysates. In Drp1 IPs, treatment with CalA, a potent phosphatase inhibitor[1], increased both pDrp1^{S616} and pDrp1^{S637} immunoreactivity (lane 1 versus 2), FSK increased pDrp1^{S637} immunoreactivity due to Protein Kinase A activation[2] (lane 1 versus 3), and SST decreased both pDrp1^{S616} and pDrp1^{S637} immunoreactivity, as it is a nonspecific protein kinase inhibitor[3] (lane 1 versus 4). **(B),** Anti-Drp1 IPs and input lysates from WT and Bok KO HeLa cells were probed as indicated; Mcl-1 serves as a loading control for the input lysates. pDrp1^{S616} and pDrp1^{S637} immunoreactivity was quantified and normalized to Drp1 levels in the IPs using Image Lab software; a representative immunoblot is shown. Normalized pDrp1^{S616} and pDrp1^{S637} immunoreactivity in Bok KO cells (lanes 3 and 4) was 101 $\pm$ 12 % and 120 $\pm$ 31% of that seen in WT cells (lanes 1 and 2), respectively (mean $\pm$ SEM, n=3). An unpaired t-test with Welch’s correction was used to determine significance; p=0.96 and p=0.58, respectively.

References

[1] Ishihara, H., Martin, B. L., Brautigan, D. L., Karaki, H., Ozaki, H., Kato, Y., Fusetani, N., Watabe, S., Hashimoto, K., Uemura, D., and et al. (1989) Calyculin A and okadaic acid: inhibitors of protein phosphatase activity, *Biochem Biophys Res Commun 159*, 871-877.
[2] Laurenza, A., Sutkowski, E. M., and Seamon, K. B. (1989) Forskolin: a specific stimulator of adenylyl cyclase or a diterpene with multiple sites of action?, *Trends Pharmacol Sci 10*, 442-447.
[3] Ruegg, U. T., and Burgess, G. M. (1989) Staurosporine, K-252 and UCN-01: potent but nonspecific inhibitors of protein kinases, *Trends Pharmacol Sci 10*, 218-220.
