# Supplementary material for: Identification of the Bok Interactome Using Proximity Labeling
Source: Front Cell Dev Biol. 2021 May 31;9:689951. doi: 10.3389/fcell.2021.689951 (PMC8201613; doi:10.3389/fcell.2021.689951)
Supplement: Supplementary file 11 [file Data_Sheet_7.PDF]

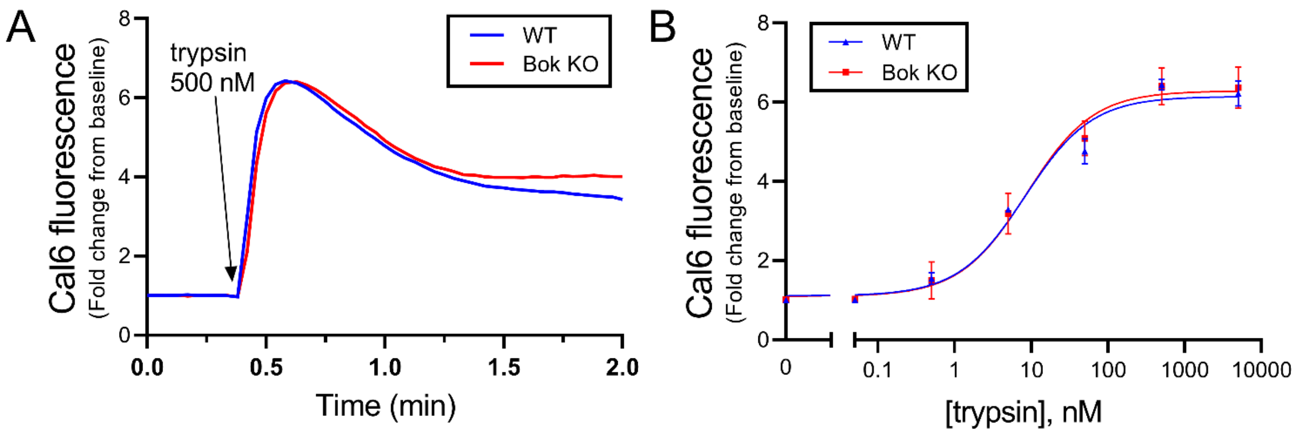

Supplementary Figure 7. G-protein-coupled receptor-mediated Ca^{2+} signaling in WT and Bok KO HeLa cells. Cells were set into 96-well plates and the following day the Cal6-based FLIPR Calcium Assay was performed as described[1]. **(A),** Temporal response to trypsin; traces shown are the average of triplicate wells for an individual representative experiment. **(B),** Trypsin dose-response curves (mean ± SEM, n=3). Data was graphed using GraphPad Prism software.

References

[1] Szczesniak, L. M., Bonzerato, C. G., Schulman, J. J., Bah, A., and Wojcikiewicz, R. J. H. (2021) Bok binds to a largely disordered loop in the coupling domain of type 1 inositol 1,4,5-trisphosphate receptor, *Biochem Biophys Res Commun 553*, 180-186.
