# Supplementary material for: Identification of the Bok Interactome Using Proximity Labeling
Source: Front Cell Dev Biol. 2021 May 31;9:689951. doi: 10.3389/fcell.2021.689951 (PMC8201613; doi:10.3389/fcell.2021.689951)
Supplement: Supplementary file 12 [file Data_Sheet_8.PDF]

Supplementary Methods

Sample processing for mass spectrometry

Excised gel lanes were subjected to in-gel trypsin digestion[1]. Briefly, gel pieces were washed with 50 mM ammonium bicarbonate in 50% acetonitrile, reduced with DTT, alkylated with iodoacetamide, washed again, and impregnated with 75 µL of 5 ng/µL trypsin (Trypsin Gold; Promega) solution overnight at 37°C. Peptides were extracted using solutions of 50% and 80% acetonitrile with 0.5% formic acid, and the recovered solution dried down in a vacuum concentrator.

Dried peptides were dissolved in 60 µL of 0.1% trifluoroacetic acid, and desalted using 2-core MCX stage tips (3M)[2]. The stage tips were activated with acetonitrile followed by 3% acetonitrile with 0.1% trifluoroacetic acid. Next, samples were applied, followed by two washes with 3% acetonitrile with 0.1% trifluoroacetic acid, and one wash with 65% acetonitrile with 0.1% trifluoroacetic acid. Peptides were eluted with 75 µL of 65% acetonitrile with 5% NH_4OH, and dried.

Liquid chromatography-mass spectrometry

Samples were dissolved in 25 µL of water containing 2% acetonitrile and 0.5% formic acid. 2 µL of this solution was injected onto a pulled tip nano-LC column with 75 µm inner diameter packed to 25 cm with 3 µm C18AQ particles, pore size 120 Å (Dr. Maisch). The peptides were separated using a gradient from 3 – 28% acetonitrile over 60 min, followed by a 7 min ramp to 85% acetonitrile. The column was connected in line with an Orbitrap Lumos via a nanoelectrospray source operating at 2.2 kV. The mass spectrometer was operated in data-dependent top speed mode with a cycle time of 2.5s. MS1 scans were collected at 6×10^4 resolution with AGC target of 6.0×10^5 and maximum injection time of 50 ms. HCD fragmentation was used followed by MS2 scans in the Orbitrap at 1.5×10^4 resolution with AGC target of 1.0×10^4 and 100 ms maximum injection time.

Database searching

MS data were searched using SequestHT in Proteome Discoverer (version 2.4, Thermo Scientific) against the human proteome from Uniprot, containing 70709 sequences, concatenated with common laboratory contaminant proteins. Enzyme specificity for trypsin was set to semi-tryptic with up to 4 missed cleavages. Precursor and product ion mass tolerances were 10 ppm and 0.6 Da, respectively. Cysteine carbamidomethylation was set as a fixed modification. Methionine oxidation and lysine biotinylation were set as a variable modification. The output was filtered using the Percolator algorithm with strict FDR set to 0.01.

Protein characterization

Protein characterization, including sub-cellular localization and function, was assessed using UniProt [3] and GeneCards[4]. Localization categories were nucleus, ER, mitochondria, Golgi, endosome/lysosome/peroxisome, cytoskeleton, cytosol, and PM, with some proteins assigned multiple (up to 3) localizations. Protein lists from different TurboID constructs were compared to each other using Venny 2.1 (Figure 2A, 3B, Supplementary Figure 4, available at https://bioinfogp.cnb.csic.es/tools/venny/).

References

[1] Shevchenko, A., Tomas, H., Havlis, J., Olsen, J. V., and Mann, M. (2006) In-gel digestion for mass spectrometric characterization of proteins and proteomes, *Nat Protoc 1*, 2856-2860.

[2] Rappsilber, J., Ishihama, Y., and Mann, M. (2003) Stop and go extraction tips for matrix-assisted laser desorption/ionization, nanoelectrospray, and LC/MS sample pretreatment in proteomics, *Anal Chem 75*, 663-670.

[3] UniProt, C. (2021) UniProt: the universal protein knowledgebase in 2021, *Nucleic Acids Res 49*, D480-D489.

[4] Stelzer, G., Rosen, N., Plaschkes, I., Zimmerman, S., Twik, M., Fishilevich, S., Stein, T. I., Nudel, R., Lieder, I., Mazor, Y., Kaplan, S., Dahary, D., Warshawsky, D., Guan-Golan, Y., Kohn, A., Rappaport, N., Safran, M., and Lancet, D. (2016) The GeneCards Suite: From Gene Data Mining to Disease Genome Sequence Analyses, *Curr Protoc Bioinformatics 54*, 1 30 31-31 30 33.
